# The physiological role of the brain GLP-1 system in stress

**DOI:** 10.1080/23312025.2016.1229086

**Published:** 2016-09-14

**Authors:** Marie K. Holt, Stefan Trapp

**Affiliations:** ^a^ Centre for Cardiovascular and Metabolic Neuroscience, Department of Neuroscience, Physiology & Pharmacology, University College London, WC1E 6BTLondon, UK; ^b^ Imperial College London, UK

**Keywords:** glucagon-like peptide-1, preproglucagon neurons, stress, food intake, hypothalamus-pituitary-adrenal axis, sympathetic nervous system

## Abstract

Glucagon-like peptide-1 (GLP-1) within the brain is a potent regulator of food intake and most studies have investigated the anorexic effects of central GLP-1. A range of brain regions have now been found to be involved in GLP-1 mediated anorexia, including some which are not traditionally associated with appetite regulation. However, a change in food intake can be indicative of not only reduced energy demand, but also changes in the organism’s motivation to eat following stressful stimuli. In fact, acute stress is well-known to reduce food intake. Recently, more research has focused on the role of GLP-1 in stress and the central GLP-1 system has been found to be activated in response to stressful stimuli. The source of GLP-1 within the brain, the preproglucagon (PPG) neurons, are ideally situated in the brainstem to receive and relay signals of stress and our recent data on the projection pattern of the PPG neurons to the spinal cord suggest a potential strong link with the sympathetic nervous system. We review here the role of central GLP-1 in the regulation of stress responses and discuss the potential involvement of the endogenous source of GLP-1 within the brain, the PPG neurons.

## 1. GLP-1 is a regulator of homeostasis

Glucagon-like peptide-1 (GLP-1) is an incretin and neuropeptide best known for its role in glucose homeostasis and appetite regulation (Holst, 2007; Kreymann, Ghatei, Williams, & Bloom, 1987; Tang-Christensen et al., 1996; Turton et al., 1996; Wang et al., 1995). In the periphery, GLP-1 is released from *L* cells in the gut following ingestion of food (Vilsbøll et al., 2003). From the blood it reaches the pancreas where it acts on β-cells to enhance the secretion of insulin in response to glucose while inhibiting the release of glucagon from α-cells (de Heer, Rasmussen, Coy, & Holst, 2008; Holst, 2007; Ørskov, Holst, & Nielsen, 1988; Vilsbøll, Krarup, Madsbad, & Holst, 2003). In addition, peripheral GLP-1 has proliferative and protective effects on islet cells and inhibits gastric emptying (Egan, Bulotta, Hui, & Perfetti, 2003; Farilla et al., 2002; Nauck et al., 1997). Central GLP-1, here defined as GLP-1 acting within the central nervous system (CNS), is well-established as a potent regulator of food intake (Barrera et al., 2011; Larsen, Tang-Christensen, & Jessop, 1997; Turton et al., 1996; Williams, Baskin, & Schwartz, 2009). Within the brain, GLP-1 is produced in a subset of granule cells or short axon cells of the olfactory bulb, some pyramidal cells of the piriform cortex and a few neurons in the lumbar-sacral spinal cord (Larsen, Tang-Christensen, Holst, & Ørskov, 1997; Merchenthaler, Lane, & Shughrue, 1999; Thiebaud et al., 2016; Zheng, Cai, & Rinaman, 2015). However, the primary source of GLP-1 in the brain is in preproglucagon (PPG) neurons in the nucleus tractus solitarii (NTS) and the intermediate reticular nucleus in the lower brainstem (Merchenthaler et al., 1999). PPG neurons, also referred to as GLP-1 neurons, particularly in studies on rat, project throughout the brain to autonomic control centres and this projection pattern largely matches the expression of GLP-1 receptors in the brain (Larsen, Tang-Christensen, Holst, et al., 1997; Llewellyn-Smith, Gnanamanickam, Reimann, Gribble, & Trapp, 2013; Llewellyn-Smith, Reimann, Gribble, & Trapp, 2011; Merchenthaler et al., 1999; Trapp & Cork, 2015; Vrang, Hansen, Larsen, & Tang-Christensen, 2007). PPG neurons are activated *in vitro* and *in vivo* by a range of satiety hormones and peripheral signals relating to food intake and general homeostasis (Hisadome, Reimann, Gribble, & Trapp, 2010, 2011; Merchenthaler et al., 1999; Rinaman, 1999b; Trapp & Richards, 2013).

It is clear that central GLP-1 reduces food intake. What is less clear is the physiological purpose and cause of this anorexic response. Importantly, a reduction in food intake can be a response to not only decreased energy demand, but also to changes in the emotional state that reduces the motivation to eat, or to visceral malaise leading to reduced appetite. Early studies addressed the role of taste aversion and nausea in the regulation of food intake. NTS GLP-1 neurons were activated by intraperitoneal injection of LiCl, a compound which is well known to cause malaise and taste aversion (Rinaman, 1999b). Furthermore, blockade of the GLP-1 receptor using the antagonist Exendin (9-39) reversed the LiCl-induced suppression of appetite in rat, suggesting a role for central GLP-1 in the response to malaise (Rinaman, 1999a). However, these results could not be reproduced in mouse (Lachey et al., 2005), suggesting that subtle, but important species differences may exist in the central GLP-1 system. In another early attempt to anatomically dissect different GLP-1 actions in the brain, van Dijk and Thiele (1999) demonstrated that bilateral lesions in the PVN prevented the induction of satiety by GLP-1, but not the conditioned taste aversion observed with GLP-1. They also showed that lesions in the amygdala prevented GLP-1 induced taste aversion, but rats retained the GLP-1 induced reduction in food intake. These experiments clearly demonstrated the existence of two separate pathways for GLP-1 effects on satiety and malaise. As further evidence for a role of GLP-1 in the response to general malaise, there is now data supporting a link between inflammation and GLP-1 mediated reduction in food intake and the cytokine interleukin-6 (IL-6) has been shown to activate PPG neurons in the NTS (Anesten et al., 2016; Shirazi et al., 2013). More recent studies have revealed that GLP-1 receptor signalling in the mesolimbic system affects food intake by modulating reward pathways (Dickson et al., 2012; Mietlicki-Baase et al., 2013, 2014) and that activation of GLP-1 receptors in the hippocampus, a region not traditionally associated with appetite regulation, reduces food intake (During et al., 2003; Hsu, Hahn, Konanur, Lam, & Kanoski, 2015).

From all these emerging targets it is becoming evident that GLP-1 in the brain does not simply inhibit metabolically driven food intake, but that the reduced appetite may be part of a wider response to emotional stress or visceral malaise (Ghosal, Myers, & Herman, 2013; Kinzig et al., 2003; Kreisler & Rinaman, 2016; Maniscalco, Kreisler, & Rinaman, 2012; Maniscalco, Zheng, Gordon, & Rinaman, 2015; Rinaman, 1999b). In this review we discuss the evidence for the involvement of central GLP-1 in the regulation of stress and consider the potential role of the central source of GLP-1, the PPG neurons. Most evidence has been gathered in mouse and rat and since few anatomical and functional differences have been observed between species (including human and non-human primates), we assume here that most findings are relevant across species (Vrang & Grove, 2011; Zheng et al., 2015), though any paradigms where conflicting evidence exists between species, will be highlighted. We begin by briefly describing the organism’s response to stress. In the next section, we review the evidence for a role for GLP-1 in the regulation of stress responses and finally we discuss the potential role of the central source of GLP-1, the PPG neurons, in the stress response.

## 2. Stress activates two parallel coping systems, the hypothalamic-pituitary axis and the sympathetic nervous system

**Figure 1.**
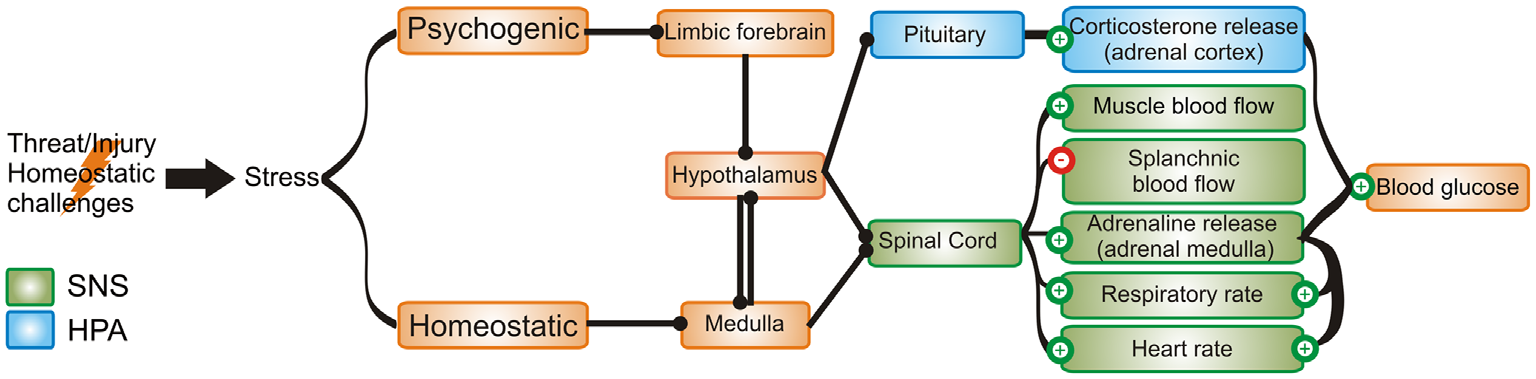
Stressors activate forebrain and brainstem regions to prepare for “fight-or-flight”.

Notes: Acute injury or a perceived threat leads to activation of psychogenic and/or homeostatic stress. Psychogenic stress recruits areas in the limbic areas of the forebrain, including the hippocampus and the amygdala, which influence the activity of hypothalamic nuclei. A homeostatic challenge activates brainstem nuclei, which either relay this information to hypothalamic regions or directly to sympathetic preganglionic neurons in the spinal cord. The hypothalamus receives modulatory input from forebrain and medullary nuclei and is the main driver of the HPA axis (in blue) and the sympathetic nervous system (SNS, in green). Release of corticotropin-releasing hormone (CRH) from parvocellular neurons in the paraventricu lar nucleus of the hypothalamus on cells expressing adrenocorticotropic hormone (ACTH) leads to release of ACTH into the bloodstream via the blood, ACTH reaches the adrenal cortex where it stimulates the release of corticosterone (cortisol in humans). Corticosterone mobilises glucose by increasing gluconeogenesis and by counteracting the effects of insulin. Parallel activation of the SNS (green) leads to release of adrenalin from postganglionic sympathetic neurons onto target tissues, including blood vessels, the adrenal medulla, heart and the respiratory system. This leads to increased muscle blood flow and decreased blood flow to the organs in the abdominal cavity (splanchnic blood flow) ensuring sufficient oxygen and energy supply to the muscles. The secretion of adrenaline from the adrenal medulla into the blood further increases heart and respiratory rate and the mobilisation of glucose through glycogenolysis and lipolysis.

Stress is defined as the collection of physiological responses to homeostatic (physical) and psychological (perceived) challenges (Dayas, Buller, Crane, Xu, & Day, 2001; Sawchenko, Li, & Ericsson, 2000; Ulrich-Lai & Herman, 2009). Stress allows the organism to cope with aversive stimuli and appropriate stress responses are essential to the survival of the organism. On the other hand, inappropriate chronic stress responses can lead to long-term damaging disorders such as anxiety and depression. Neural control of stress responses is complex and involves forebrain, brainstem and spinal cord circuits that ultimately converge to activate two important effectors in the body’s response to stress: the sympathetic part of the autonomic nervous system (SNS) and the hypothalamus-pituitary-adrenal (HPA) axis (Ulrich-Lai & Herman, 2009) producing the so-called “fight-or-flight” response (Figure 1). A perceived threat to homeostasis and the well-being of the organism leads to rapid activation of the SNS. Heart and blood pressure are increased via recruitment of catecholaminergic neurons in the rostral ventrolateral medulla (RVLM), the raphe pallidus and sympathetic preganglionic neurons in the spinal cord as well as higher order autonomic control sites in the hypothalamus and amygdala (Dampney, 1994; Dayas et al., 2001; Dimicco & Zaretsky, 2007). Increased sympathetic activity leads to release of adrenaline from the adrenal medulla and adrenaline in turn increases cardiac output and respiratory rate while redirecting blood flow to skeletal muscle and mobilising glucose from liver and skeletal muscle (Ulrich-Lai & Herman, 2009). The parallel recruitment of the HPA axis involves the activation of parvocellular neurons in the paraventricular nucleus of the hypothalamus (PVN). These neurons release corticotrophin-releasing hormone (CRH) onto adrenocorticotropic hormone (ACTH) expressing neurons in the anterior pituitary. From the pituitary, ACTH is released into the bloodstream through which it reaches the cortex of the adrenal gland. In the adrenal cortex ACTH elicits release of corticosterone, which works to mobilise glucose by stimulating gluconeogenesis securing the body’s demand for glucose during homeostatic challenges (Ulrich-Lai & Herman, 2009). Corticosterone provides negative feedback at the level of the pituitary and the hypothalamus to limit HPA activity (Ulrich-Lai & Herman, 2009).

## 3. GLP-1 activates both the HPA axis and the sympathetic nervous system

The majority of evidence for a role of GLP-1 in stress has been gathered using supraphysiological doses of GLP-1 or GLP-1 analogues (typically Exendin-4) to activate GLP-1 receptors both peripherally and centrally. These studies have shown that recruitment of central GLP-1 receptors potently activates the HPA axis in both humans and rodents with a resulting increase in both ACTH and corticosterone/cortisol concentrations in blood (Gil-Lozano et al., 2010; Kinzig et al., 2003). The adrenal cortex does not express GLP-1 receptors and isolated cells from the adrenal glands do not release corticosterone in response to GLP-1 ruling out the possibility that GLP-1 acts directly on the adrenal cortex (Dunphy, Taylor, & Fuller, 1998; Gil-Lozano et al., 2014). Importantly, central administration of Exendin-4 leads to an increase in corticosterone in rodents, suggesting an involvement of central GLP-1 receptors possibly expressed on CRH expressing neurons in the hypothalamus (Gil-Lozano et al., 2014; Larsen, Tang-Christensen, & Jessop, 1997; Sarkar, Fekete, Légrádi, & Lechan, 2003). In fact, central blockade of CRH receptors blocks Exendin-4-induced increases in ACTH and corticosterone, establishing a role for central GLP-1 receptors in HPA axis regulation (Gil-Lozano et al., 2014).

In a study investigating the involvement of central GLP-1 in both homeostatic and psychogenic stress, Kinzig et al. (2003) found that injections of GLP-1 into the PVN increased blood ACTH and corticosterone concentrations. Targeting the amygdala increased anxiety-like behaviour with animals spending significantly less time in the open arms of an elevated plus maze after infusion of GLP-1. Different types of stressors, i.e. homeostatic vs. psychogenic, are known to activate distinct neural pathways and the data described above suggests that GLP-1 regulates both homeostatic and psychogenic stress responses through distinct neural pathways (Dayas et al., 2001; Kinzig et al., 2003).

Intriguingly, activation of central GLP-1 receptors not only stimulates the HPA axis, but also appears to increase sympathetic activity, the other important pathway for the physiological response to stress (Smits et al., 2016; Yamamoto et al., 2002). This is measured as an increase in heart rate in both rodents and humans following GLP-1 receptor activation (Gil-Lozano et al., 2014; Griffioen et al., 2011; Robinson, Holt, Rees, Randeva, & O’Hare, 2013; Smits et al., 2016; Yamamoto et al., 2002). Central GLP-1 receptor activation was found to stimulate both autonomic regulatory neurons, neurons in the spinal cord and cells in the adrenal medulla signifying clear recruitment of the sympathetic nervous system (Yamamoto et al., 2002). This suggests that central GLP-1 could activate both arms of the stress response, the HPA axis and the sympathetic nervous system.

## 4. NTS GLP-1 neurons are ideally situated to integrate signals of stress

Studies using supraphysiological activation of GLP-1 receptors provide little information about the physiological role of the endogenous GLP-1 system in stress regulation. Exogenous activation of central GLP-1 receptors clearly triggers release of ACTH and corticosterone, but the question remains, whether there is an endogenous source of GLP-1 eliciting these responses under physiological conditions? Further, which signals trigger release of GLP-1 from that source? GLP-1 released from L cells in the gut is rapidly degraded in the liver and bloodstream making it unlikely that GLP-1 reaches receptors in the brain in large quantities (Deacon, 2004; Hansen, Deacon, Ørskov, & Holst, 1999; Holst & Deacon, 2005; Kieffer, McIntosh, & Pederson, 1995; Vilsboll, Krarup, Deacon, Madsbad, & Holst, 2001). In contrast, the central source of GLP-1, the PPG neurons, are ideally situated in the NTS in the caudal brainstem to receive and process signals of stress from the rest of the body (Kreisler & Rinaman, 2016; Maniscalco et al., 2012; Merchenthaler et al., 1999; Rinaman, 1999b; Vrang, Phifer, Corkern, & Berthoud, 2003). The NTS is a well-established central site of integration of visceral afferent signals concerning general homeostasis, which are relayed to higher brain centres (Grill & Hayes, 2012). From the NTS, PPG neurons project to autonomic control sites throughout the brain including the PVN, the dorsomedial hypothalamus and the RVLM, which are all involved in the control of the HPA axis and/or sympathetic activity (Larsen, Tang-Christensen, Holst, et al., 1997; Llewellyn-Smith et al., 2011, 2013; Vrang et al., 2007). In the PVN, there is dense expression of GLP-1 receptors and GLP-1 immunoreactive axons make contact with parvocellular CRH producing neurons, supporting a role for PPG neurons in the regulation of CRH secretion from the hypothalamus (Cork et al., 2015; Larsen, Tang-Christensen, Holst, et al., 1997; Sarkar et al., 2003).

NTS neurons are generally thought to regulate sympathetic activity indirectly through ascending projections to either the RVLM or hypothalamic nuclei. Recent findings in our laboratory have demonstrated that PPG neurons in the brainstem not only send ascending projections to autonomic control sites mainly in the hypothalamus, but also directly innervate sympathetic preganglionic neurons in the spinal cord (Llewellyn-Smith et al., 2015). These data highlight the possibility that the central GLP-1 system may integrate incoming stress signals and relay them via both ascending projections to CRH neurons in the PVN to elicit HPA activity and descending projections to preganglionic sympathetic neurons in the intermediolateral column (IML) and central autonomic area (CAA) of the spinal cord to increase sympathetic outflow.

In a thorough dissection of HPA-GLP-1 crosstalk, Lee et al. (2016) explored the neural pathways underlying GLP-1 receptor initiated increases in corticosterone in rat. Systemic (intraperitoneal; i.p.) exendin-4 activated catecholaminergic (CA), non-PPG neurons in the NTS and RVLM. Most of these neurons were found to project to the parvocellular and magnocellular PVN. Selective ablation of this CA-PVN connection using DBH-saporin prevented the i.p. exendin-4 induced increase in blood concentrations of corticosterone. This demonstrated that activation of the HPA axis by systemic exendin-4 is dependent on CA input to the PVN. On first sight this seems surprising given that PPG neurons project heavily to the PVN and make contacts to CRH neurons as discussed above. However, keeping in mind that PPG neurons do not express GLP-1 receptors, these findings might just indicate that the peripheral and central GLP-1 systems are more separate than widely thought. It also emphasises that still more studies are needed that explore which exact peripheral signals activate PPG neurons and which do not.

## 5. The central GLP-1 system is activated in response to both homeostatic and psychogenic stress

An early study suggesting a link between central GLP-1 and stress was conducted by Rinaman (1999b). Interoceptive stress was induced through intraperitoneal injection of LiCl. LiCl is considered a nauseogenic agent and is known to reduce food intake while increasing the concentration of stress hormones ACTH and corticosterone in the blood (Kinzig, Hargrave, & Honors, 2008). LiCl was found to activate GLP-1 neurons in the NTS which were found to project to the HPA-regulating parvocellular region of the PVN (Rinaman, 1999b). Kinzig et al. later demonstrated that the LiCl-induced increase in stress hormones is dependent on central GLP-1 signalling (Kinzig et al., 2003). Third ventricular infusion of a GLP-1 receptor antagonist abolished the increase in both ACTH and corticosterone following systemic LiCl injections. The discovery of close appositions from GLP-1 immunoreactive axon terminals on CRH producing PVN neurons in rat further substantiate these findings (Tauchi, Zhang, D’Alessio, Stern, & Herman, 2008). Similarly, axons of mouse PPG neurons have close appositions on CRH producing PVN neurons (personal communication, Ida Llewellyn-Smith). These data suggest that homeostatic stress following a toxic challenge activates the central GLP-1 system to recruit systemic stress pathways.

Similarly, acute psychogenic stress induced by physical restraint reduces food intake and activates the HPA axis (Kinzig et al., 2008). Maniscalco et al. recently demonstrated that the number of cFOS positive GLP-1 neurons increases following 30mins restraint stress or 5mins exposure on an elevated platform, suggesting that GLP-1 neurons are activated by psychogenic stress (Maniscalco et al., 2015). Furthermore, they found that 30 mins restraint stress reduced food intake at the onset of dark phase and that this hypophagic response was blocked by central infusion of GLP-1 receptor antagonist, suggesting that psychogenic stress recruits the central GLP-1 system to reduce food intake (Maniscalco et al., 2015).

Finally, in a study focusing on the role of GLP-1 in cocaine addiction, GLP-1 neurons were found to be activated by an injection of corticosterone into the fourth ventricle (Schmidt et al., 2016). Fourth ventricle corticosterone reduced cocaine self-administration and this reduction was blocked by GLP-1 receptor antagonism in the ventral tegmental area (Schmidt et al., 2016). These data suggest that not only does central GLP-1 activate the HPA axis, but corticosterone in turn activates the central GLP-1 system.

## 6. Conclusions

It is clear that the central GLP-1 system plays a role in the regulation of food intake. However, increasing numbers of studies report effects of GLP-1 in brain regions not classically associated with appetite control and it is becoming increasingly clear that central GLP-1 may be responsible for much wider homeostatic control. In particular, the anorexic effects of GLP-1 may in some cases be secondary to responses to homeostatic and psychogenic stress.

**Figure 2.**
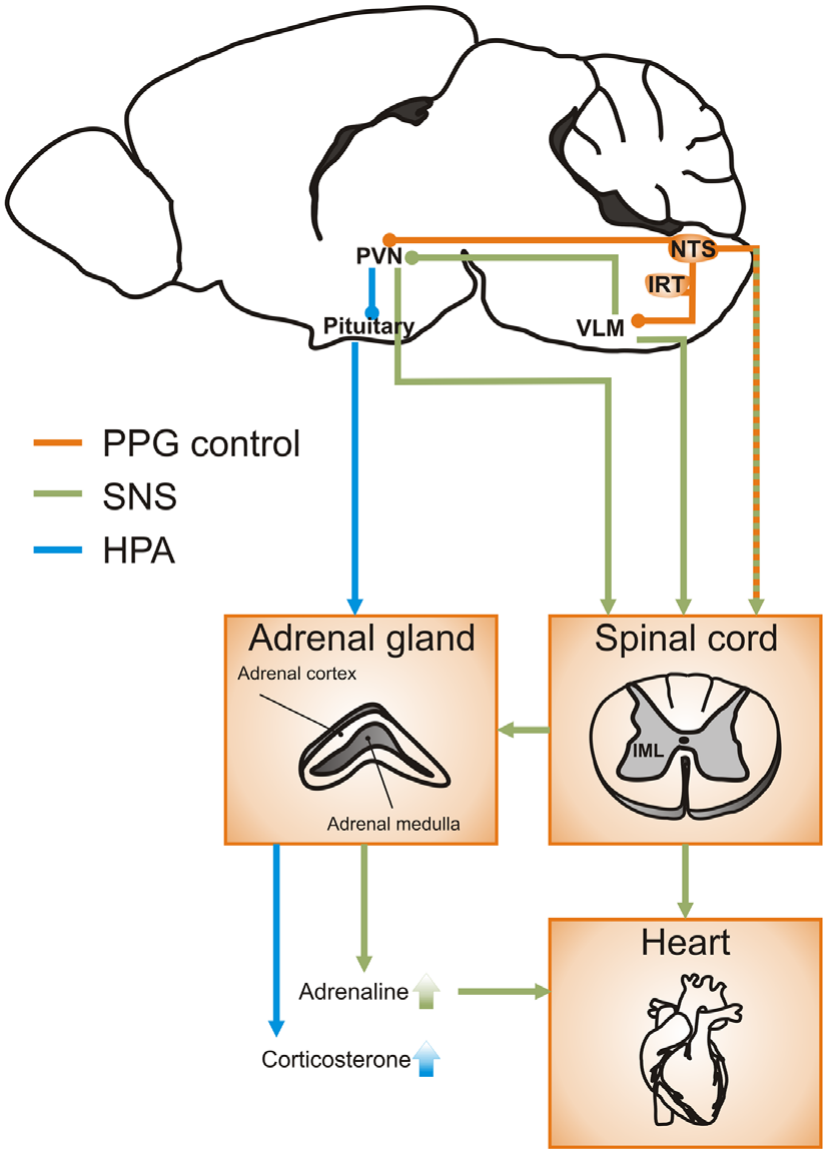
PPG pathways to activate both HPA axis and sympathetic nervous system in the control of stress responses.

Notes: PPG neurons in the nucleus of the solitary tract (NTS) and the intermediate reticular nucleus (IRT) are activated by stressors. Activation of PPG neurons leads to release of GLP-1 (orange) onto parvocellular cells in the PVN, which in turn activate the HPA axis (blue arrows) by stimulating cells in the pituitary to release adrenocorticotropic hormone (ACTH). ACTH acts on the adrenal cortex to increase the secretion of corticosterone. In parallel, PPG neurons send descending axons directly to spinal sympathetic preganglionic neurons in the intermediolateral column (IML) and ascending axons to presympathetic neurons in VLM and PVN. Recruitment of PVN and VLM neurons also leads to activation of the sympathetic nervous system (green arrows) via descending fibres to the sympathetic preganglionic neurons in the IML in the spinal cord, which in turn increase heart rate and stimulate the release of adrenaline from the adrenal medulla.

We have discussed here evidence for a role of central GLP-1 in the regulation of the body’s stress response. It is clear that overactivation of brain GLP-1 receptors enhances secretion of stress hormones and activity of the SNS. In contrast, the role of the central source of GLP-1, the PPG neurons, is less explored and the neural pathways underlying the GLP-1 mediated modulation of stress are largely unknown. The evidence discussed here suggests a model in which peripheral signals of homeostatic and psychogenic stress activate PPG neurons (Figure 2). The PPG neurons are ideally situated in the NTS to integrate signals of stress and relay that signal on to parvocellular neurons in the PVN. Furthermore, in this model psychogenic or homeostatic stress would lead to release of GLP-1 from PPG neurons. Downstream activation of GLP-1 receptors then increases sympathetic activity via direct and indirect pathways. Directly, PPG neurons modulate the activity of sympathetic preganglionic neurons in the spinal cord. Indirectly, GLP-1 from PPG neurons activates presympathetic RVLM and PVN neurons which in turn project to spinal sympathetic preganglionic neurons.

The evidence discussed here suggests that central GLP-1 does not simply regulate food intake in response to changes in energy demand, but that the PPG neurons are activated by stress and that central GLP-1 modulates acute stress, homeostatic or psychogenic, by increasing corticosterone, mobilising glucose and increasing heart rate, allowing the organism to cope with potential threats.

## Public Interest Statement

Glucagon-like peptide-1 analogues are arguably the most promising new drugs in the fight against diabetes and obesity. In order to fully understand the therapeutic potential as well as the associated risks for these drugs, a thorough understanding of the effects of GLP-1 in the brain has to be obtained. This review focuses on one important aspect, the potential role of central GLP-1 in stress responses.
